# Supplementary material for: Negative regulation of TH17-mediated inflammation by the nuclear receptor REV-ERBβ
Source: iScience. 2026 Oct 15;29(11):117577. doi: 10.1016/j.isci.2026.117577 (PMC13691276; doi:10.1016/j.isci.2026.117577)
Supplement: Supplementary file 1 [file mmc1.pdf]

iScience, Volume *29*

Supplemental information

Negative regulation of T_H17-mediated inflammation by the nuclear receptor REV-ERBβ

Sean Campbell, Sarah A. Mosure, Mohammed Amir, Jonathan Chuck, Qun Lu, and Laura A. Solt

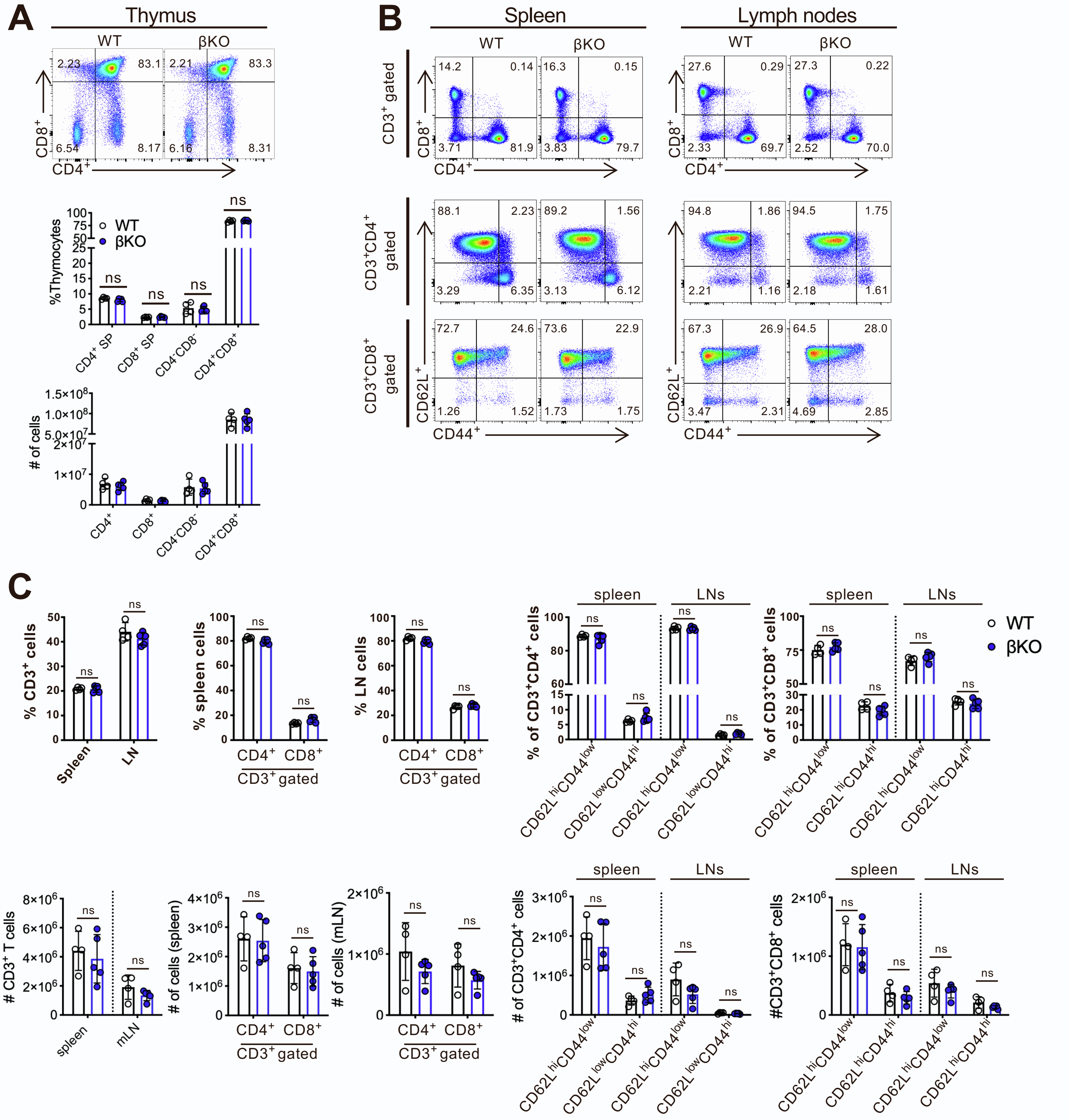

Figure S1. **Characterization of REV-ERBβ KO mice.** (**A**) Thymocytes from 8-week old, male REV-ERBβ wild-type (WT) and REV-ERBβ knock-out (βKO) mice stained with anti-CD4 and anti-CD8 antibodies and analyzed using flow cytometry. Graphs (below) depicts the frequency and numbers of thymocytes per subset. (SP – single positive; DP – double positive; DN – double negative) (**B**) (*top panels*) FACS analysis of $CD3^+CD4^+$ or $CD3^+CD8^+$ T cells in the spleen and lymph nodes of WT or βKO mice. (*bottom panels*) FACS analysis of naïve ($CD62L^{high}CD44^{low}$) and effector T cell ($CD62L^{low}CD44^{high}$) populations from the spleen and lymph nodes of WT or βKO mice. (**C**) Graphs depicting the frequencies and numbers of T cells and their subsets in the spleens and lymph nodes of WT or βKO mice shown in Panel B. Data represent mean±s.e.m and 2 separate experiments. (n=4-5/group). ns, not significant ($p>0.05$).

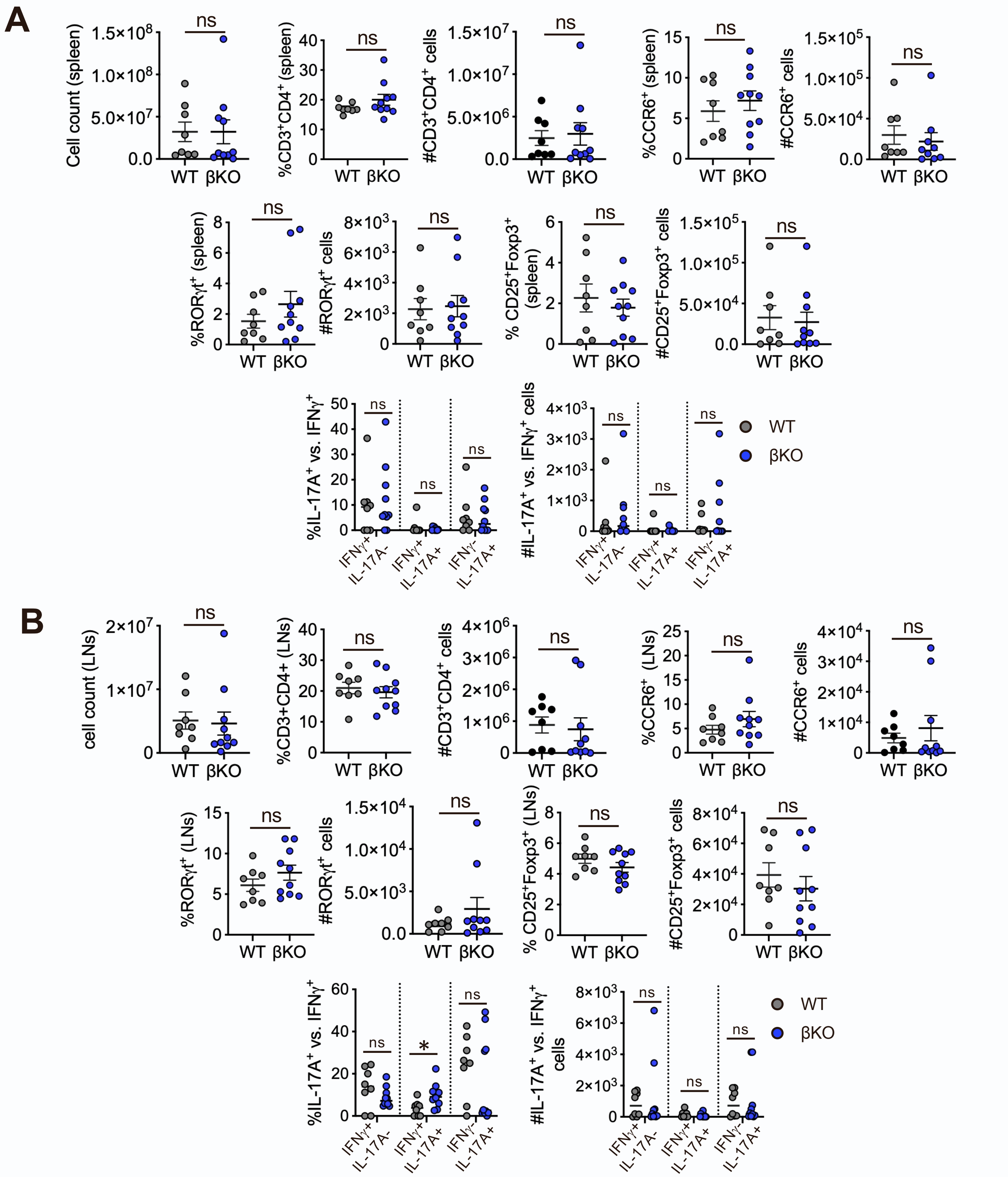

Figure S2. **REV-ERBβ-deficiency does not affect T cell populations in the spleen or dLNs.** Graphs summarizing percent (%) and total cell numbers of WT vs. βKO $CD3^+CD4^+$ T cells, $CD4^+CCR6^+$ cells, $ROR\gamma t^+$ cells, $CD25^+Foxp3^+$ cells, and $IL\text{-}17A^+IFN\gamma^-$, $IL\text{-}17A^+IFN\gamma^+$, and $IL\text{-}17A^-IFN\gamma^+$ cells in the **(A)** spleens and **(B)** draining lymph nodes (LNs) at peak of disease (Day 15). Cells were gated on live, $CD45^+CD3^+CD4^+$ cells. Each symbol represents an individual female mouse. (n=8/WT, n=10/βKO). Data represents mean±s.e.m. Female mice were used. Student's *t*-tests were performed for statistical analysis. *$*p<0.05$,* ns = not significant.

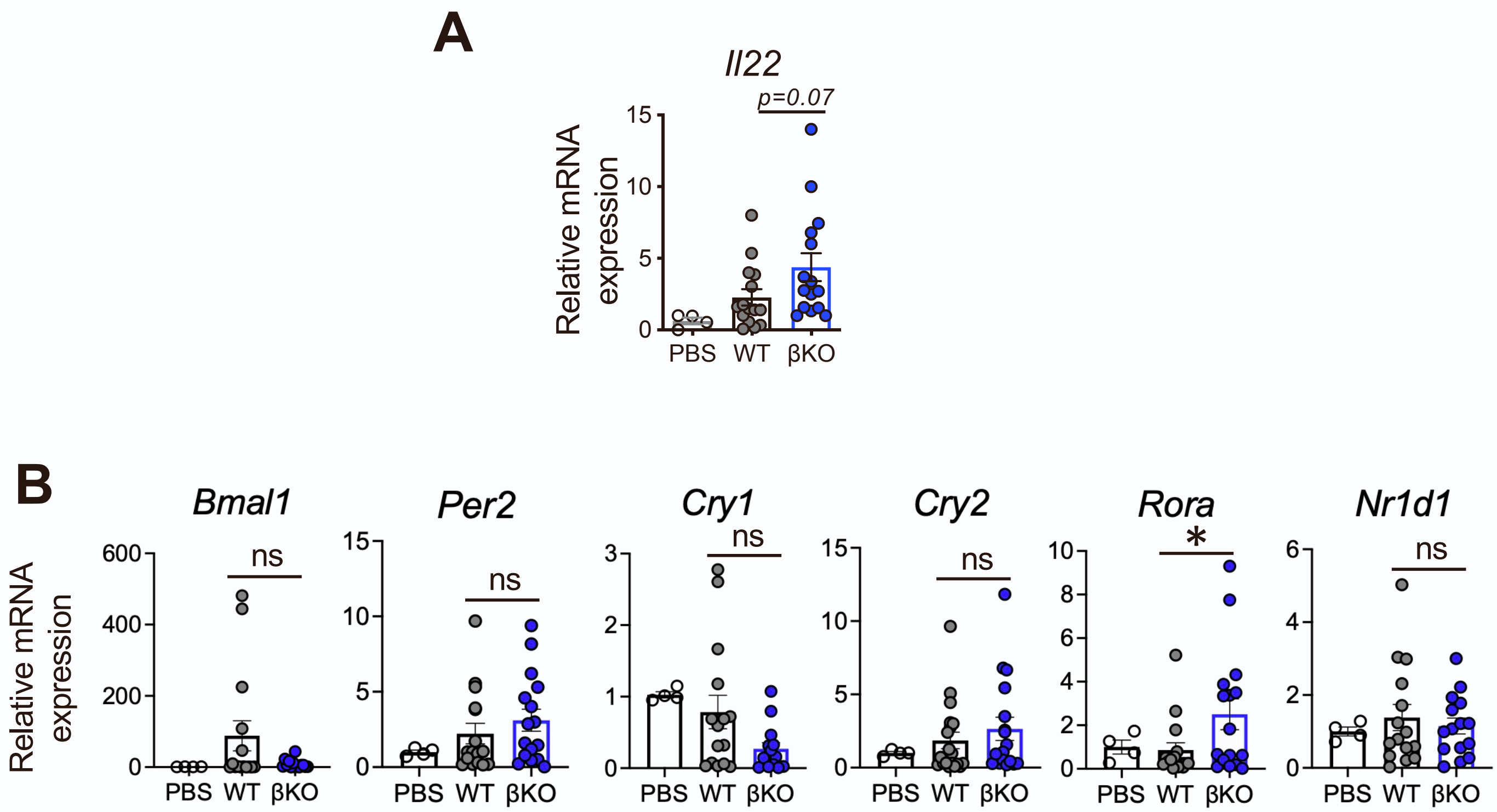

<u>Figure S3</u>. REV-ERBβ-deficiency leads to enhanced IL-22 expression *in vivo*. qRT-PCR analysis of **(A)** *Il22* expression or **(B)** circadian gene expression in the proximal colon from *Rag1*$^{-/-}$ mice receiving PBS, WT, or βKO CD4^{+} T cells. *18s* was used as the internal control. Each symbol represents an individual female mouse. (n=13 for WT, n=15 for βKO, and n=4 for PBS). Data represents mean±s.e.m. Student's *t*-tests were performed for statistical analysis.

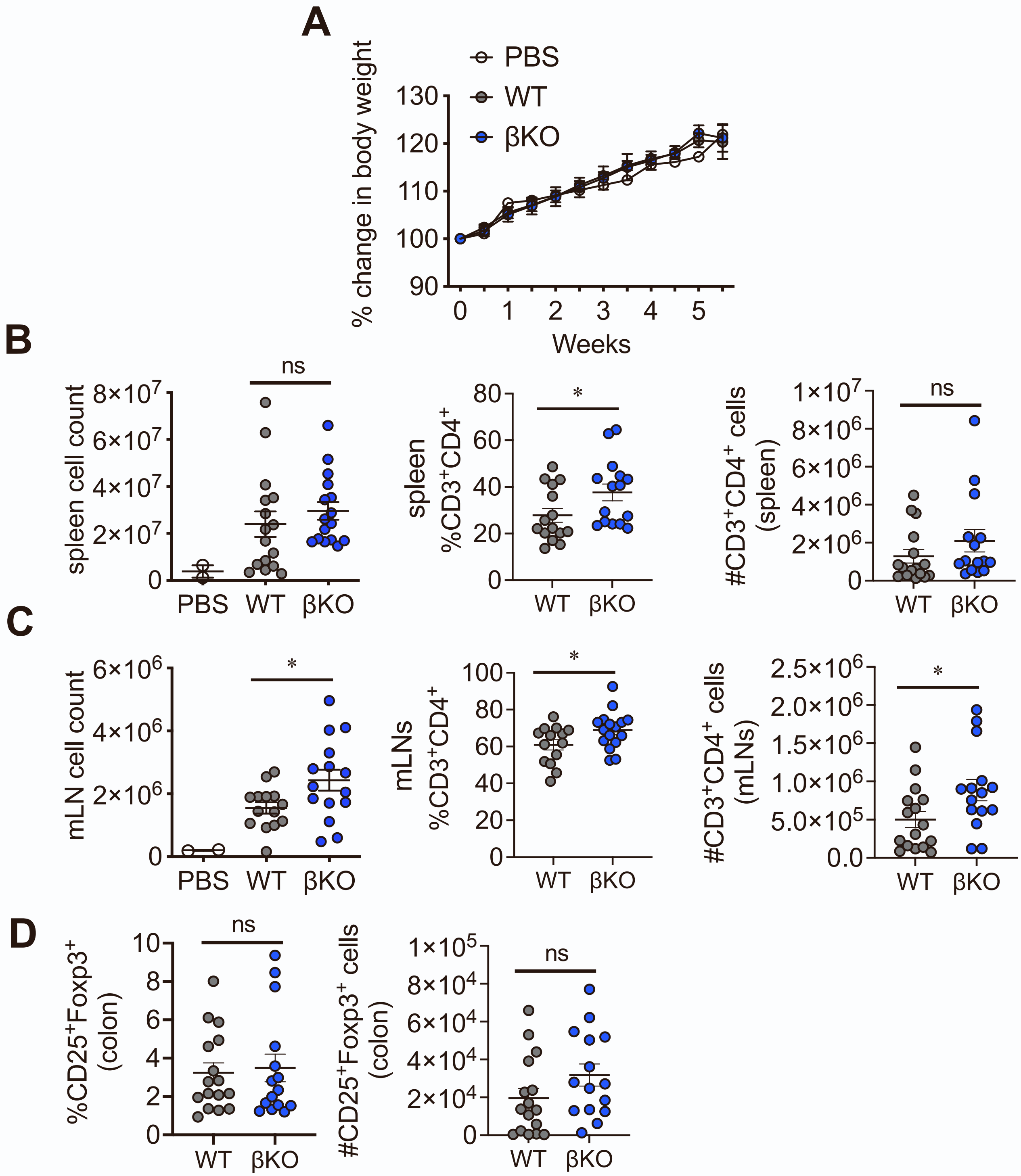

<u>Figure S4</u>. Analysis of REV-ERBβ-deficient T cells at peak of disease. (A) Graph depicts percent change in body weight of *Rag1*$^{-/-}$ mice receiving REV-ERBβ wild-type (WT), knock-out (βKO) T cells, or control (no cells, PBS) for the short-term (6 week) experiment. **(B)** Graphs summarizing number of cells found in the spleen (*left*), frequency, and number of $CD3^+CD4^+$ T cells found in the spleens (*middle, right*, respectively) of *Rag1*$^{-/-}$ mice receiving WT, βKO T cells, or PBS after 6 weeks. **(C)** Graphs summarizing number of cells found in the mLNs (*left*), frequency, and number of $CD3^+CD4^+$ T cells found in the mLNs (*middle, right*, respectively of *Rag1*$^{-/-}$ mice receiving WT, βKO T cells, or PBS after 6 weeks. **(D)** Graphs summarizing % and number of $Foxp3^+$ T regulatory cells found in the of *Rag1*$^{-/-}$ mice receiving WT and βKO T cells after 6 weeks. Each symbol represents an individual female mouse. (n=14 for WT, n=15 for βKO, and n=2 for PBS). Data represents mean±s.e.m. Two-way ANOVA (body weight) and Student's *t*-tests were performed for statistical analysis. *$p<0.05$; ns, not significant.

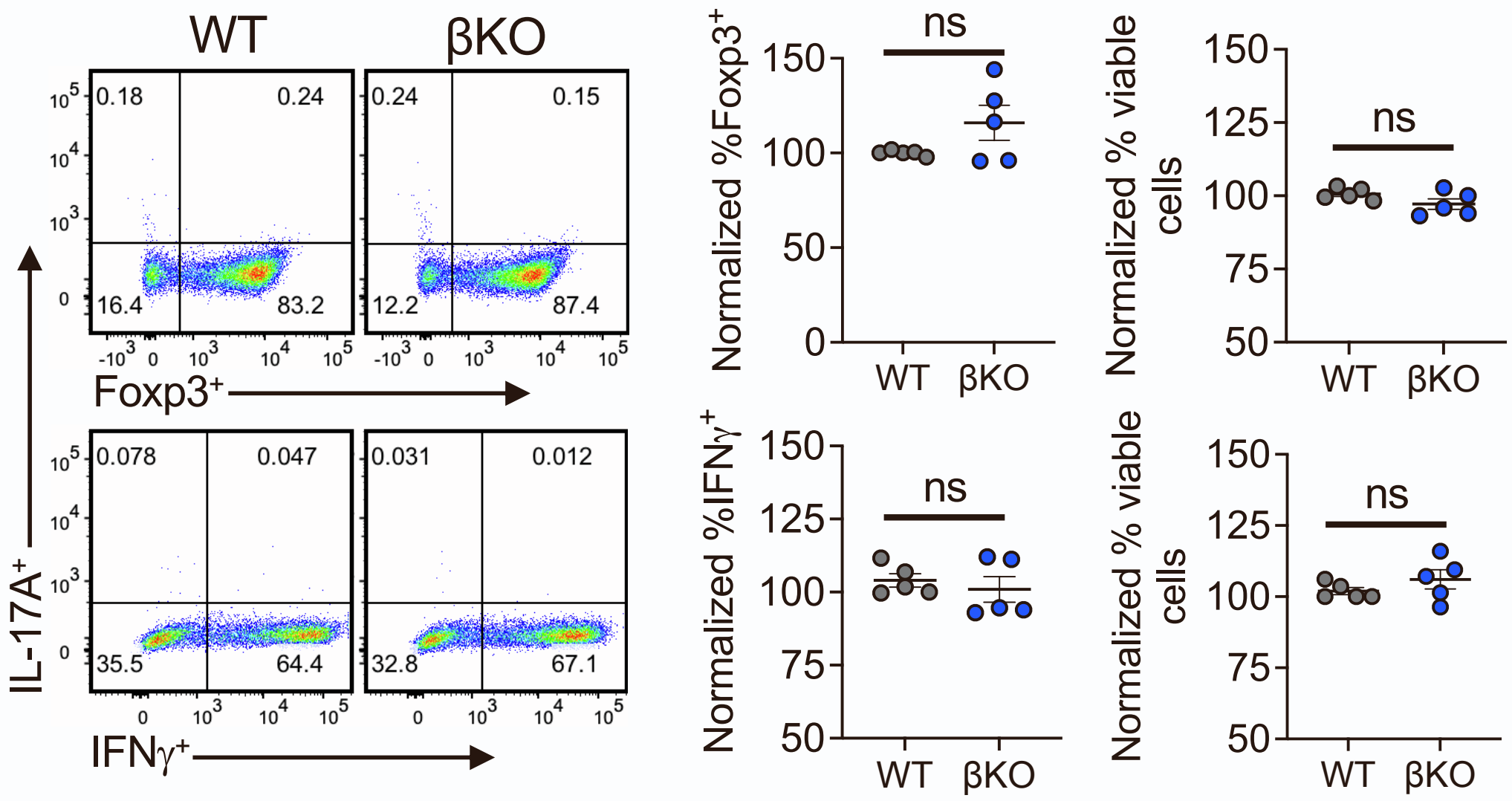

<u>Figure S5</u>. Loss of REV-ERBβ does not affect the development of T_H1 and iTreg cells *in vitro*. FACS analysis of naïve CD4⁺ T cells differentiated into T_H1 or iTreg cells from WT or βKO mice. FACS plots show IL-17A, IFNγ, and Foxp3 expression in the cultures. Graphs to the right show normalized IFNγ or Foxp3 expression as well normalized viability in all cell cultures (n=5). Statistics determined using Student's *t*-test. ns, not significant.

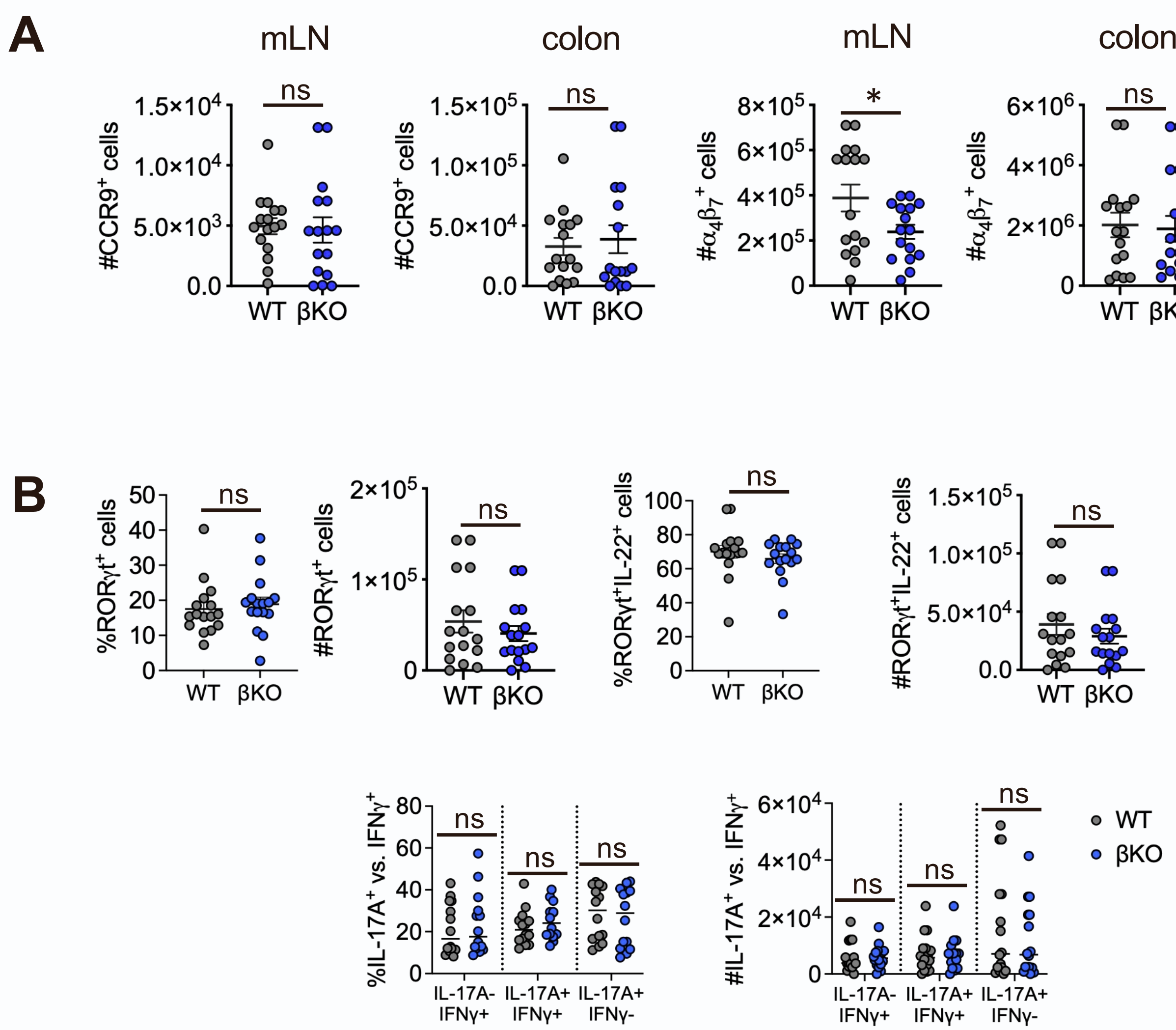

<u>Figure S6</u>. Loss of REV-ERBβ does not affect percent and numbers of cells population in the mLNs. (A) Graphs summarizing the numbers of CCR9⁺ and α4β7⁺ cells in mLN and colon of *Rag1*$^{-/-}$ recipient mice. **(B)** Graphs depicting the frequency and numbers of RORγt expression, IL-22 expression, and IL-17A⁺IFNγ⁻, IL-17A⁺IFNγ⁺, and IL-17A⁻IFNγ⁺ WT and βKO cells in the mLNs of *Rag1*$^{-/-}$ recipient mice at 6 weeks post-transfer. Cells were gated on live, CD3⁺CD44⁺CD4⁺ and CC45⁺ or CD45⁻ cells. Each symbol represents an individual female mouse [n=16 (WT), n=16 βKO)]. Data are presented as mean values ±s.e.m. Data are representative of two combined independent experiments. Statistics determined using Student's *t*-test. $*p<0.05$, ns, not significant.

Table S1. qRT-PCR primers used to assess gene expression

Gene name	Forward primer (5' - 3')	Reverse Primer (5' - 3')
b-actin	CCACAGCTGAGAGGGAAATC	AAGGAAGGCTGGAAAAGAGC
18s	GTAACCCGTTGAACCCCATT	CCATCCAATCGGTAGTAGCG
Cxcl10	TCCTTGTCCTCCCTAGCTCA	ATAACCCCTTGGGAAGATGG
Ifng	TGGCTGTTTCTGGCTGTTACT	GCTCTGCAGGATTTTCATGTC
Il6	CATGTTCTCTGGGAAATCGTG	TCCAGTTTGGTAGCATCCATC
Il17a	CTCCAGAAGGCCCTCAGACTAC	AGCTTTCCCTCCGCATTGACACAG
Il17f	GAGGATAACACTGTGAGAGTTGAC	GAGTTCATGGTGCTGTCTTCC
Il22	TCA TCG GGG AGA AAC TGT TC	CAT GTA GGG CTG GAA CCT GT
Nfil3	CTTTCAGGACTACCAGACATCCAA	GATGCAACTTCCGGCTACCA
Nr1d1	ACCTTTGAGGTGCTGATGGT	CTCGCTGAAGTCAAACATGG
Nr1d2	TGTGAAAACAGGCAAAACCA	CCCTTACAGCCTTCACAAGC
Rorc	CCGCTGAGAGGGCTTCAC	TGCAGGAGTAGGCCACATTACA
Tnfa	TCAGCCGATTTGCTATCTCAT	TGGAAGACTCCTCCCAGGTAT
